# Co-involvement of stimulants with opioids in North America: A 'silent epidemic'

**DOI:** 10.1371/journal.pmen.0000319

**Published:** 2025-07-16

**Authors:** Yutong Li, Derek V. Pierce, Shelly Vik, Kathryn Dong, Scott Patten, Yanbo Zhang, Jake Hayward, Andrew J. Greenshaw, Bo Cao

**Affiliations:** 1 Department of Psychiatry, University of Alberta, Edmonton, Alberta, Canada; 2 Department of Community Health Sciences, Cumming School of Medicine, University of Calgary, Calgary, Canada; 3 Department of Emergency Medicine, Faculty of Medicine & Dentistry, University of Alberta, Edmonton, Canada; 4 Department of Computing Science, University of Alberta, Edmonton, Alberta, Canada; University of Western Australia, AUSTRALIA

## Abstract

The opioid epidemic unfolded in three distinct waves, with the latest comprising of deaths attributed to illegally manufactured synthetic opioids. We discuss evidence for a 'silent epidemic' alongside the opioid epidemic comprising co-ingestion of stimulants including methamphetamine and cocaine. Data regarding opioid- and stimulant-use trends (specifically methamphetamine and cocaine) were collected from the National Institute on Drug Abuse (NIDA) (1999–2021) in the United States for unintentional and intentional deaths. For Canada, opioid- (2016–2022) and stimulant-use (2018–2022) trends for unintentional and intentional deaths were collected from the Public Health Agency of Canada and the Alberta Substance Use Surveillance System. Joinpoint regression analysis was used to investigate trends for opioid and stimulant use in the United States and Canada. As a proxy for public interest in opioid- and stimulant-related trends, Google Trends Relative Search Interest (RSI) was used to measure the interest over time (2004-present) for opioid- and stimulant-related search terms. Spearman's non-parametric correlation was used to measure the relationship between the RSI and drug-related deaths. Although public attention is mostly directed at the opioid epidemic, stimulant-use is increasing year over year. Moreover, the use of stimulants and opioids together is associated with a higher rate of increase in drug-related deaths each year compared to stimulants or opioids alone. Despite the importance of the potential stimulant contribution to opioid-involved deaths, Google Trends RSI revealed public interest in stimulants has dropped from a peak value in 2004–2005, whereas relative interest in opioids is relatively much higher on Google in comparison to the interest in stimulants. Given the contribution of stimulants to the ongoing drug poisoning crisis there is an urgent need to develop and implement strategies that reduce the health risks associated with stimulant use. To achieve this, we must raise awareness among clinicians, policymakers and the general public regarding the potential impacts of stimulant use, including deliberate and unaware co-use of stimulants and opioids, on drug poisoning deaths, population health and the healthcare system.

## Introduction

There were 80,411 and 7,993 opioid-involved deaths in 2021 in the United States and Canada, respectively [1,2], each one representing a loss to families and communities. Furthermore, opioid-involved deaths and opioid use disorder have led to a $1.02 trillion loss in the United States economy in 2017 due to the loss of life, medical costs, and burden on the justice system [3]. Interestingly, the opioid epidemic is localized to North America, where 15 per 100,000 deaths were attributed to opioid overdose, whereas in Europe, 1.2 deaths per 100,000 were attributed to overdose in 2017 [4]. Throughout the last two decades, the opioid crisis has experienced three waves, with an increasing number of deaths from each wave [5]. The first wave was defined by the increase rates of prescription opioid use in the 1990s for pain management [5,6]. The second and third waves were driven by an increase in deaths from heroin, and synthetic opioids (e.g., fentanyl), respectively. More recently, the intentional or unintentional co-involvement of opioids and stimulants is contributing to increase in overdose deaths [7].

Methamphetamine and cocaine, two of the most commonly used stimulants, were linked to a third of overdose deaths in 2017 in the United States [8–10]. Notably, stimulants were associated with a high number of secondary substance use disorder diagnoses for emergency and in-patient department encounters, which was associated with a higher healthcare usage cost [11]. This leads to higher healthcare costs for in-patient and emergency encounters for stimulant use. Despite the impact of stimulants on the healthcare system, public attention has largely been focused on combating opioid overdose deaths. However, we cannot ignore the importance of the contribution of stimulants to the opioid epidemic, as 22.3% of people with an opioid use disorder regularly used amphetamines [12]. Furthermore, a high percentage of people with a primary stimulant use disorder also used opioids, as 72.7% of cocaine-related deaths involved opioids [13]. Considering the high percentage of individuals that use both stimulants and opioids, it is important to understand the impact of stimulants in the context of the opioid crisis, particularly when the co-involvement of stimulants with opioids are touted as a 'silent' or 'twin' epidemic [14–17]. Furthermore, studies have highlighted the important role of stimulants in opioid-involved deaths because individuals that use both opioids and stimulants have twice the hazard of overdose compared to individuals who only use opioids [16].

Because of the severity of the 'silent' epidemic, we must monitor the future progress of this epidemic through investigating the public attention on opioids and stimulants, which can influence policymakers' prioritization in combating this issue. Google Trends can be utilized to accomplish this goal. Google Trends is a free tool provided by Google that allows users to research daily interest in different search terms and topics across various geographical areas [18]. Specifically, Google Trends measures the relative search interest (RSI), or the normalized data of the search interest for a specific topic or term. For example, an RSI of 100 indicates a maximum search interest for a term at the specified location and period [19]. It has been used in the context of monitoring the opioid crisis to determine demand for substance use disorder treatment from the healthcare system [20]. Furthermore, proposals have also been made to utilize Google Trends to make predictions on methamphetamine-related crimes in central Europe, and as a monitoring tool for the opioid crisis [21,22]. Thus, Google Trends can be used as a proxy to measure public interest in topics such as the opioid and stimulant crisis.

Given the severity of opioid- and stimulant-related deaths in North America, there is a necessity to understand the impact of the co-involvement of opioids and stimulants on death rates, especially when the public is not fully aware of the stimulant involvement in the opioid crisis. Furthermore, increasing public attention and support regarding drug-related issues can increase willingness to invest in improving substance use-related outcomes [23]. The aim of this study is to describe relevant trends for opioid and stimulant overdose deaths in North America and to determine whether trends in public interest and attention mirror those trends.

## Methods

### Data usage

United States data related to drug overdose death trends from 1999-2021 were extracted from the National Institute on Drug Abuse, a publicly available data source [2]. Opioid overdose deaths were identified using ICD-10 codes T40.0-T40.4, and T40.6. Prescription opioids and synthetic opioids were identified using the codes T40.2-T40.3, and T40.4, respectively. Stimulants were defined as T43.6 and T40.5. The nature of the opioid- and stimulant-related deaths were unintentional and intentional, as defined by the ICD-10 codes drug poisoning (X40–X44), suicide (X60-X64), homicide (X85), poisoning of undetermined intent (Y10–Y14).

Canadian data on substance use trends from 2016-2022 were obtained from the Public Health Agency of Canada and the Alberta Substance-Use Surveillance System, publicly available data sources [24,25]. The stimulant-related deaths data from the Public Health Agency of Canada (2018–2022) did not incorporate deaths from Alberta. Hence, the stimulant-related deaths from the Alberta Substance-Use Surveillance System were incorporated into the stimulant-related death age-adjusted rates from the Public Health Agency of Canada following the conversion into age-standardized rates using the protocol outlined by Statistics Canada [26]. The United States 2000 standard population was used to adjust Canadian opioid and stimulant data to ensure that both U.S. and Canadian trends are comparable [27–29]. We retrieved estimates for the demographic population data used to support the conversion into age-adjusted rates from Statistics Canada [30]. Furthermore, we calculated the age-adjusted rate for the co-involvement of stimulants and opioids in Canada, as there is no calculated age-adjusted rate for the co-involvement of opioids and stimulants in the Public Health Agency of Canada data. Age-adjusted rates were represented as rate per 100,000 population. This was completed by multiplying the percent co-involvement of opioids with stimulants in the Public Health Agency data by the age-adjusted rate for opioid-involved deaths in Canada. Opioid- and stimulant-involved deaths of an intentional and unintentional nature from the Public Health Agency of Canada were collected by Chief Coroners/Chief Medical Examiners from each province and territory. For the Alberta Substance-Use Surveillance System, unintentional opioid- and stimulant-related poisonings are determined by Alberta's Office of the Chief Medical Examiner.

### Search interest using Google Trends

Google Trends was used to determine search interest for opioids and psychostimulants [31]. Search terms used for opioid-involved search terms are: “opioids” + “heroin” + “fentanyl” + “oxycontin” + “oxycodone” + “codeine” + “hydrocodone” + “morphine” + “carfentanil”, where the “+” sign denotes OR [20,32]. Because methamphetamine and cocaine are among the most widely used psychostimulants of concern, we used search terms relating to these substances. Furthermore, we accounted for other common spellings of the drug in addition to the dictionary terms for each drug (e.g., methamphetamines and meth) [20]. To account for research term results relating to references to popular culture and media, we used the “-“symbol to exclude extraneous search terms, and topics. The search terms we used are cocaine - bear + “crack cocaine”, and “methamphetamines + methamphetamine + meth + amphetamine. We also selected “health” for the search category to ensure that extraneous topics are not captured within the search trends. Examples of extraneous topics include movie-, popular culture-, and other related topics that were popular at the time of the Google Trends RSI query (e.g., the movie Cocaine Bear).

### Statistical analysis

Joinpoint Trend Analysis Software (referred as Joinpoint) was used to analyze the trends, and joinpoints/inflection points within the opioid- and stimulant-related death trends [33]. Specifically, Joinpoint was used to analyze the annual percent change (APC) in the opioid and stimulant-related trends, which can be interpreted as the percent increase per year within a time range (e.g., an APC of 1% in 2012 means an increase of 1% between the years 2011–2012). Because the null hypothesis for the APC is 0, a statistically significant APC signals that the APC is different from zero at an alpha of 0.05. Furthermore, a 95% confidence interval was provided for each of the APC of the fitted model for both Canada and the United States. The outcome of the analysis was the age-adjusted death rate for opioid- and stimulant-related deaths in the United States and Canada. The year was defined as the independent variable. Because the age-adjusted death rates do not provide standard errors, homoscedasticity or constant variance was assumed for the analysis. The model selection method was the Weighted Bayesian Information Criterion (WBIC), a model selection method that determines the weighted average of the Bayesian Information Criterion (BIC) and the BIC3 [34,35]. Specifically, the WBIC serves to choose the number of joinpoints within a fitted model. This method was chosen because it is considered to perform well in different scenarios with small and large effect sizes [34,35].

We first assessed the normality of the Google Trends, and death trends using the Shapiro Wilk test of normality. Because the data were not normally distributed, we used Spearman's non-parametric correlation, to determine the correlation between the search interest using Google Trends, and the opioid- and stimulant-related death trends in the United States. Correlation for the Canadian opioid- and stimulant-related death trends were not computed because of insufficient data, as the Public Health Agency of Canada started data collection in 2016.

## Results

### Opioid-involved death rates in the United States and Canada

**Fig 1:**
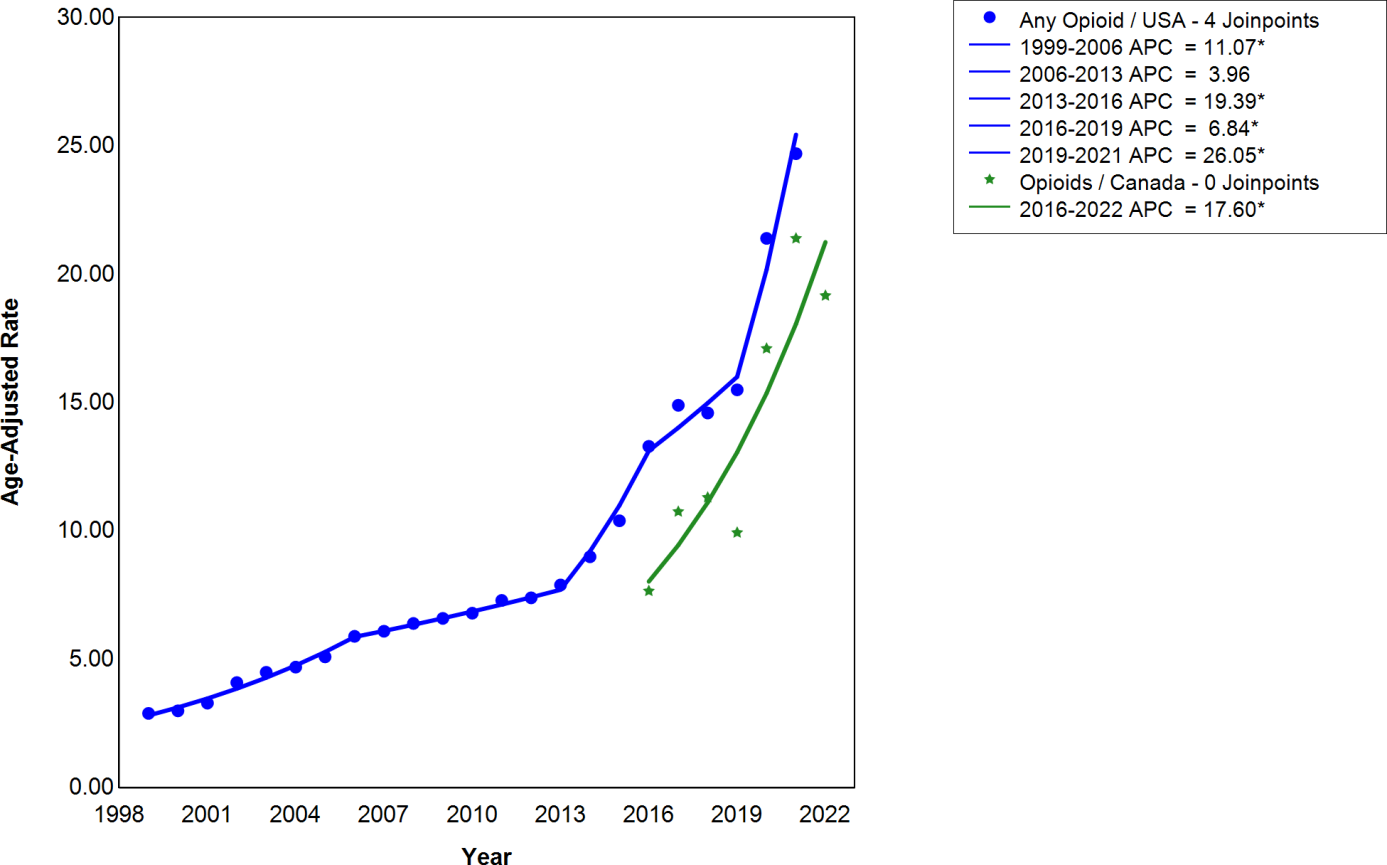
The annual percentage change (APC) in the number of deaths per year for opioid-involved deaths in the United States and Canada. Four joinpoints for the United States were constructed for 1999-2021. The APC for the United States are 11.07 (CI = 7.33 14.04), 3.96 (CI = -1.19,13.98), 19.39 (CI = 3.22,23.14), 6.84 (CI = 2.69,13.53), and 26.05 (CI = 15.55, 33.77) for 1999-2006, 2006-2013, 2013-2016, 2016-2019, and 2019-2021 respectively, where CI denotes a 95% confidence interval. For Canada, no joinpoints were constructed from 2016-2022. The APC for Canada is 17.60 for 2016-2022 (CI = 6.95, 29.99), where CI denotes a 95% confidence interval. The age-adjusted mortality rates are shown for the United States and Canada, where the deaths rates are per 100,000 population. *indicates that the APC for the specified period is statistically significant at α = 0.05.

As illustrated by Fig 1, there was an increase in opioid-involved deaths in the United States from 1999-2021. Specifically, there were four statistically significant line segments: from 1999-2006, 2013–2016, 2016–2019, 2019–2021 where the APC were 11.07 (CI = 7.33 14.04), 19.39 (CI = 3.22, 23.14), 6.84 (CI = 2.69, 13.53), and 26.05 (CI = 15.55, 33.77), respectively, where CI denotes a 95% confidence interval. The non-statistically significant segment is in in 2006–2013, where the APC was 3.96 (CI = -1.19, 13.98), as CI denotes a 95% confidence interval. Notably, there was a flattening of the opioid-involved deaths in the United States in 2006–2013, and 2016–2019, where the APCs are the lowest among the segments. For Canada, no joinpoints were constructed due to the lack of historical data. The statistically significant APC was 17.60 for 2016–2022 (CI = 6.95, 29.99), as CI denotes a 95% confidence interval.

### Stimulant-involved death rates in the United States and Canada

**Fig 2:**
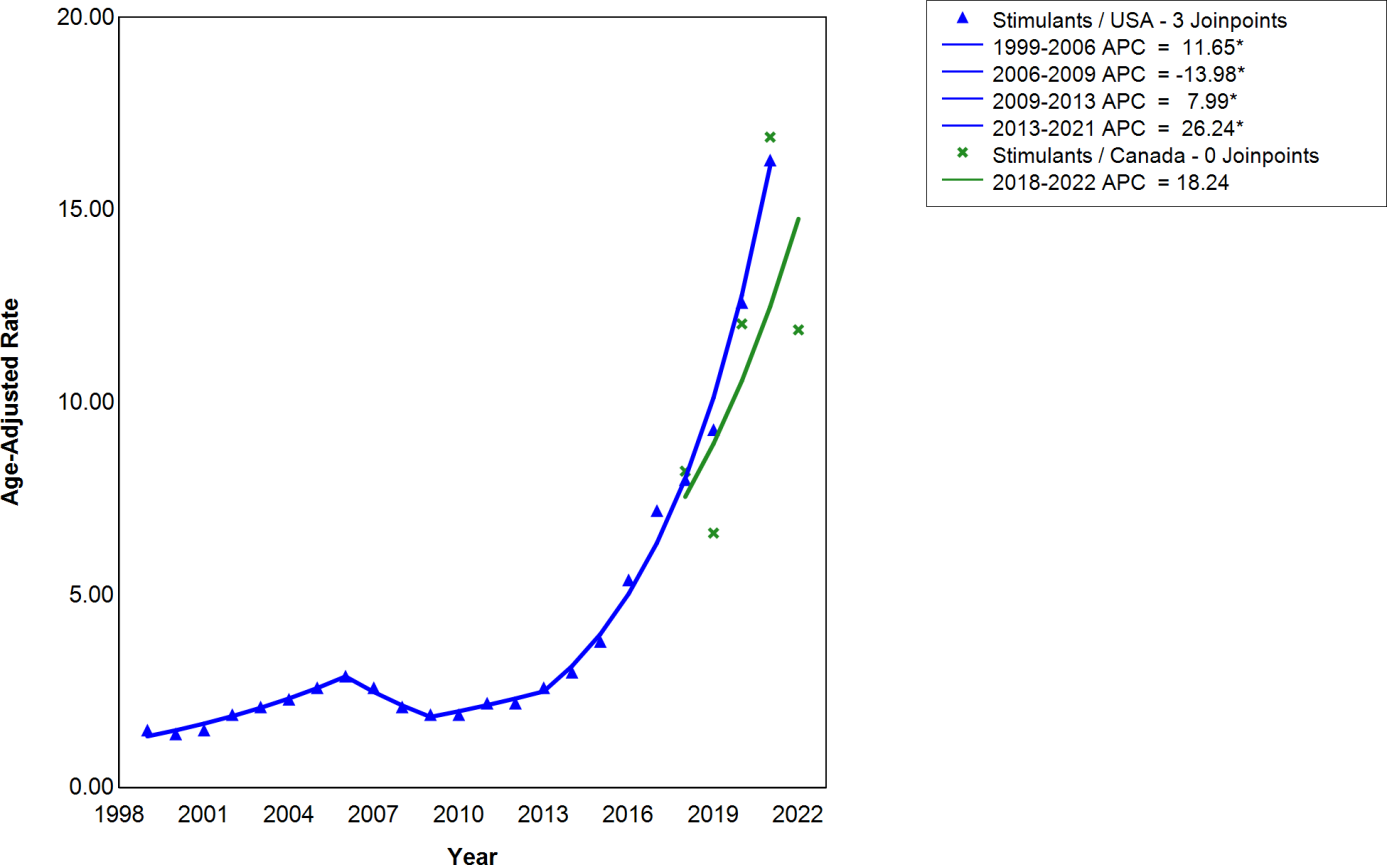
The annual percentage change (APC) in the number of deaths per year for stimulant-involved deaths in the United States and Canada. For the United States, three joinpoints are constructed for 1999-2021. The APC for the United States are 11.65 (CI = 9.52, 14.59), -13.98 (CI = -17.62, -6.14), 7.99 (CI = 0.92, 19.01), and 26.24 (CI = 24.02, 30.71) for 1999-2006, 2006-2009, 2009-2013, and 2013-2021 respectively, where CI denotes a 95% confidence interval. For Canada, no joinpoints were constructed from 2016-2022. The APC for Canada is 18.24 (CI = -16.58, 66.20) for 2018-2022, where CI denotes a 95% confidence interval. The age-adjusted mortality rates were shown for the United States and Canadian trends as per 100,000 population. * indicates that the APC for the specified period is statistically significant at α = 0.05.

There were four significant line segments for age-adjusted stimulant-involved deaths in the United States (Fig 2). The first segment was from 1999-2006, when the APC was 11.65 (CI = 9.52, 14.59). From 2006-2009, the APC was -13.98 (CI = -17.62, -6.14), signaling a decrease in stimulant-involved deaths in the USA. From 2009-2013, the APC was 7.99 (CI = 0.92, 19.01). The highest APC for the United States was from 2013-2021, at 26.24 (CI = 24.02, 30.71). In Canada, there were no joinpoints due to insufficient data. The APC in Canada from 2018-2022 was 18.24 (CI = -16.58, 66.20). CI denotes a 95% confidence interval.

### Co-involvement of opioid- and stimulant-involved death rates in the United States and Canada

**Fig 3:**
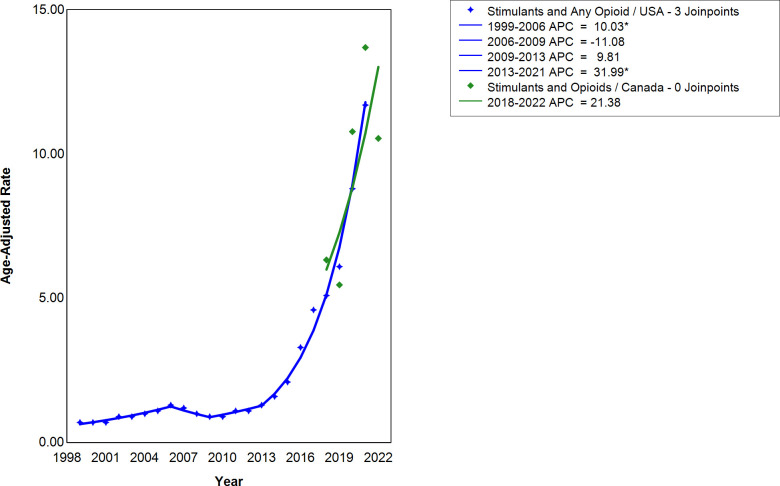
The annual percent change (APC) in the co-involvement of stimulants and opioids in the United States and Canada. Three joinpoints for the United States were constructed for 1999-2021. The APC for the United States are 10.03 (CI = 6.59, 15.20), -11.08 (CI = -15.84, 7.83), 9.81 (CI = -2.29, 28.68), and 31.99 (CI = 28.39, 41.17) for 1999-2006, 2006-2009, 2009-2013, and 2013-2021 respectively, where CI denotes a 95% confidence interval. For Canada, no joinpoints were constructed from 2018-2022. The APC for Canada is 21.38 (CI = -11.07, 64.92) for 2018-2022, where CI denotes a 95% confidence interval. Age-adjusted mortality rates are shown for the United States and Canada as per 100,000 population. * indicates that the APC for the specified period is statistically significant at α = 0.05.

There were four segments, of which two were significant, in the United States, from 1999-2021 (Fig 3). The significant segments were from 1999-2006 and 2013–2021, where the APC were 10.03 (CI = 6.59, 15.20) and 31.99 (CI = 28.39, 41.17), respectively. Non-significant segments were from 2006-2009 and 2009–2013 where the APCs were -11.08 (CI = -15.84, 7.83) and 9.81 (CI = -2.29, 28.68), respectively. Joinpoints were not available in the Canadian stimulant-involved death trend, due to insufficient data. The APC from 2018-2022 is 21.38 (CI = -11.07, 64.92). CI denotes a 95% confidence interval. In terms of the percentage co-involvement of opioids and stimulants, Canada has between 50% and 70% co-involvement, compared to 10% to 40% in the United States. Potential alternate reasons for this as a possible discrepancy may be: undercounting of opioid and stimulant co-involvement in the United States; or differential methods of recording co-involvement, or differing levels of opioid and/or stimulant contamination in the unregulated drug market.

### Google Trends interest over time for the United States and Canada show differential results for opioid and psychostimulant related search interests

**Fig 4:**
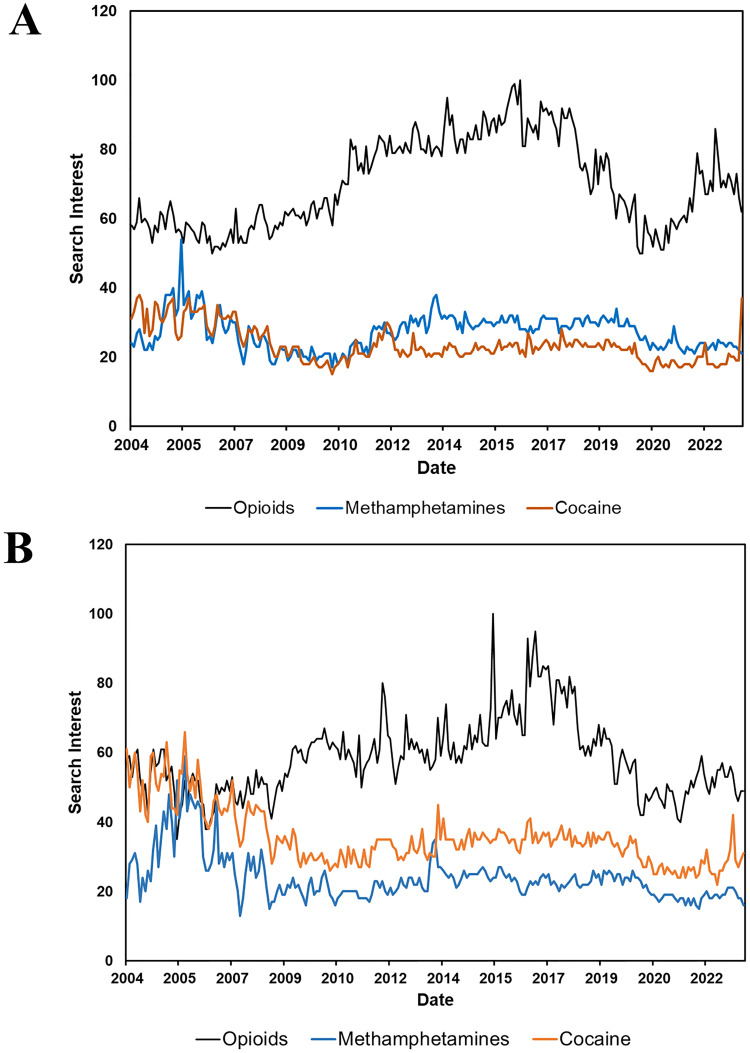
Google Trends for Stimulants (cocaine, methamphetamines) and opioid-involved search interest in the United States (A), and Canada (B). The highest search interest in a topic is denoted by 100 on the y-axis. Opioid-, methamphetamines-, and cocaine-related search terms are denoted by black, blue, and orange, respectively.

The highest search interest was in opioid-involved search terms for the United States and Canada (Fig 4). There are several peaks for opioid-involved search interest. The highest peaks of the search interest for opioids in the United States (Fig 4A) and Canada (Fig 4B) are June 2016 and August 2015, respectively. The second highest search interest peak was in 2022 for the United States and Canada, as COVID-19 exacerbated the opioid crisis. For the stimulants, the largest peaks for cocaine are April 2004 and November 2005 for the United States and Canada, respectively. Lastly, the peak for methamphetamine was August 2005 and November 2005 for the United States and Canada, respectively. Compared to the APC trends for opioid and stimulant deaths, the Google Trend RSI trends do not correlate. In comparison to stimulants, opioids have a higher RSI overall. Hence, deaths related to co-involvement of opioids and stimulants are not commensurate with the likely impact of co-involvement on population health.

## Discussion

The neglected contribution to the third wave of the opioid crisis is co-involvement of stimulants, illustrated in the data we displayed in Fig 3, reflecting a dramatic increase in the APC for the opioid and stimulant co-involvement deaths in the United States following 2013. Specifically, before and after 2013 the APC values for opioid and stimulant co-involvement death in the United States were 9.81 and 31.99, respectively (Fig 3). This increase in co-involvement was mirrored by the opioid and stimulant trend as there was a change from 3.96 to 19.39, and 7.99 to 26.24, respectively, for the same period in the United States, indicating that stimulants contributed greatly to the co-involvement trend (Figs 1 and 2). Furthermore, the higher APCs for stimulant overdose deaths signify a higher rate of growth in comparison to opioids. Canadian data indicate similar patterns in APC for opioid and stimulant deaths relative to the United States, as the APC for the opioid and stimulant co-involvement deaths was higher than either the opioid- involved or stimulant-involved deaths alone (Figs 1–3).

Because of the complex nature of the opioid crisis, there are many multifaceted contributors and causes. Proposed contributors to the alarming increases in stimulant-involved deaths include the increased availability of prescription and over the counter stimulants to adolescents due to the diversion of stimulants, increase in stimulant off-label use, lack of education for adolescents to deter risky stimulant use, increased lethality of stimulants when used in conjunction with opioids, lack of access to evidence-based treatments for stimulant-use disorder, and the contamination of illegally manufactured stimulants with other substances including opioids, among many other complex factors [15,16,36–40]. Furthermore, co-involvement may also be explained by individual-level factors. These include the individual's need to utilize the effects or synergies of opioids and stimulants (e.g., combining methamphetamine and opioids to avoid pain from multiple injections, combining stimulants and opioids to increase reinforcing effects), to seek pleasure, respond to the social context (e.g., individuals were more likely to co-use if their partner prefers co-involvement), or respond to the affordability and availability of different drugs [17,41]. The worsening of the co-involvement trends in recent years can also be attributed to the COVID-19 pandemic, where drug-use increased due to poorer mental health and economic conditions [36]. The patterns in the United States were supported by the Canadian trends as well since the APC for the co-involvement of opioids and stimulant deaths is higher than opioids or stimulants alone. To support this observation, stimulant and opioid co-involvement is a common phenomenon, as 82.4% of individuals with opioid use disorder were exposed to stimulants [36]. Finally, other contributors to the opioid- and stimulant-involved deaths relate to the potential mixing of opioids, more potent synthetic opioids with stimulants in the illegal drug market whether intentionally or unintentionally also pose a significant challenge in the opioid crisis [42].

Even though there was a continual increase in the number of deaths from the use of opioids, stimulants, and the co-involvement of opioids and stimulants, we do not see the same patterns within the Google Trends RSI. The peak of the interest was in June 2016, August 2005, and April 2004 for opioids, methamphetamines, and cocaine, respectively, in the United States. In Canada, the peak of interest was August 2015, November 2005, and November 2005 for opioids, methamphetamines, and stimulants, respectively. The differential interest in these substances is complex and unclear; however, they may lie in the history of how these crises unfolded. Specifically, for opioids, the spike that occurred around 2016 in the United States and Canada can be rooted back to the increased availability of opioids, especially synthetic opioids, around that time period and the publicized response from health authorities to combat the opioid crisis in North America [43]. In the United States, the opioid epidemic was deemed a public health emergency in 2017 [44]. Furthermore, the Canadian government announced 38 recommendations to combat the opioid crisis in 2016 [45]. In the media, more focus was placed on synthetic opioids like fentanyl, which shed light on the potency of synthetic opioids. While the opioid crisis has received extensive attention in media and from public health groups, relatively little focus has been on stimulant use. This is reflected in the lower overall Google Trends RSI for methamphetamines and cocaine in comparison to opioids. Despite the lower overall interest in stimulants compared to opioids, public awareness of stimulants did peak in 2004–2005 for the United States and Canada. The explanation for the awareness may lie in the increased production and availability of stimulants like methamphetamine and cocaine in the late 1990s- early 2000s [46,47]. The increases in stimulant availability in the early 2000s sparked increased government action, which included the Combat Methamphetamine Epidemic Act of 2005 by the United States government and the move of methamphetamine to Schedule I of the Controlled Drugs and Substances Act in Canada to enable stricter punishment for possession and trafficking methamphetamines [48,49].

Public awareness and advocacy are essential to combatting the opioid and stimulant crises, as public awareness and advocacy can result in decreased demand for substances of use, in addition to providing more interventions and support for individuals using substances [50]. Indeed, individuals who undergo education-based intervention regarding substance use are 53% less likely to use substances compared to individuals who did not undergo an intervention [51]. Hence, interventions can be designed to target individuals with polysubstance use. Furthermore, public awareness of substance use can increase social support systems in the community to prevent at-risk individuals from using substances, in addition to providing a positive environment to support individuals with substance use disorders [52,53]. Moreover, healthcare professionals and policymakers awareness of the severity of stimulant co-involvement is needed to curb overdose deaths. Given that regions with high levels of psychostimulant-involved mortality in the United States also have high opioid prescription rates, it is particularly salient to increase awareness among health care providers [54]. The covert and illegal nature of drug use can make it hard to detect and report on patterns. The unseen consumption of stimulants and opioids combined with the increasing purity and potency of unregulated drugs that are circulated discreetly, further complicate public health surveillance and intervention strategies. Stigmatization of substance use decreases public support for helping individuals with substance use disorders, which prevents resource allocation to combatting the issue [23]. Hence, strategies such as providing the public with informational fact sheets for substance use and its biological basis, motivational interviews, and positive stories of individuals recovering from substance use is essential to engage public awareness to combat stigmatization [55].Increased public awareness of the deadly impacts of opioid and stimulant co-involvement is a critical for pre-requisite for effective interventions, and preventative measures. In addition to the expanding pharmacologic treatment programs for people with opioid use disorder, more awareness and advocacy must be shed on other effective treatments such as contingency management as effective pharmacologic options for individuals with stimulant use disorder are limited [56–58].

## Limitations

Our study has five main limitations. First, we had insufficient opioid- and stimulant-involved death data from Canada as the collection process started in 2016, hence, we were not able to compare the opioid and stimulant trajectories between the United States and Canada for the period before 2016 for opioids, 2018 for stimulants, and before 2018 for the co-involvement of opioids and stimulants. Second, we were not able to account for the use of multiple substances in addition to opioid and stimulant use. For example, a common combination of polysubstance adding to opioid and stimulant co-involvement is the use of alcohol [59]. The exclusion of the analysis for the use of multiple substances was due to the absence of data from the National Institute on Drug Abuse, the Public Health Agency of Canada and the Alberta Substance-Use Surveillance System. Moreover, a significant limitation is the potential discrepancy between recorded data and actual substance use. The dynamic and covert nature of drug use patterns means that not all instances of use or overdose come to the attention of healthcare or regulatory bodies. This results in a substantive number of unreported or under-reported cases, leading to a potential underestimation of substance use prevalence and overdose incidents. Regional variations in substance use trends and reporting inconsistency are also not likely accounted for in national-level data, further complicating the issue. In addition, the continuously evolving landscape of substance use, with new synthetic drugs, contaminants, and methods of consumption, may not be immediately or adequately captured in surveillance data or in how stimulant- and opioid-deaths were determined by the Canadian and American health agencies. For example, the opioid- and stimulant-involved deaths are not mutually exclusive for the data collected by both the Canadian and American health agencies. This can impact the number of deaths recorded for individuals who use opioids and stimulants. Finally, because we are using aggregated population level data, we cannot account for other confounding factors such as socioeconomic variables or access to health care within the analysis.

## Conclusion

In conclusion, there is a substantial increase in deaths that involve both opioids and stimulants in the third wave of the opioid epidemic in North America. There is a need for increased awareness and understanding of the evolving nature of the opioid crisis and the deleterious effects of stimulant co-involvement, especially among the general population. Raising awareness is an important step toward combatting the co-involvement crisis. These findings are a call to action for public health policymakers to develop strategies for addressing both opioid and stimulant use epidemics simultaneously.

## Supporting information

S1 Table Shapiro Wilk Test of Normality results for opioid- and stimulant-involved deaths and Google Trend search interest in the United States. (DOCX)
