# Supplementary material for: Co-involvement of stimulants with opioids in North America: A 'silent epidemic'
Source: PLOS Ment Health. 2025 Jul 16;2(7):e0000319. doi: 10.1371/journal.pmen.0000319 (PMC12798603; doi:10.1371/journal.pmen.0000319)
Supplement: S1 Table — (DOCX) [file pmen.0000319.s001.docx]

S1 Table: Shapiro Wilk Test of Normality results for opioid- and stimulant-involved deaths and Google Trend search interest in the United States.

|  |  | Test Statistic | P-value |
| --- | --- | --- | --- |
| Opioids | Death | 0.8383 | 0.00557 |
|  | Google Trend | 0.8900 | 0.03836 |
| Stimulants | Death | 0.7430 | 0.0002641 |
|  | Google Trends | 0.9464 | 0.3714 |

Weak correlation between Google Trends search interest and opioid- and stimulant- related deaths

The Shapiro-Wilk test of normality for the opioid-involved deaths is shown in Table 1. Because the data are not normal, we used Spearman’s non-parametric correlation. For opioids, the Spearman’s rho was 0.3993808 with a p-value of 0.1017 for the correlation between opioid-involved deaths and opioid- related Google Trends search interest in the United States. We obtained a Spearman’s rho of 0.1842659, with a p-value of 0.4642 for stimulant-involved deaths. Because of the weak correlation, it is evident that there is weak association between the Google Trends search interest and the number of deaths involving opioid- and stimulant-use.
